# Magnetic tunnel junction made of abundant materials for memory and dynamic applications

**DOI:** 10.1038/s41598-025-20842-9

**Published:** 2025-10-09

**Authors:** Mariusz Cierpiał, Dawid Maślanka, Kacper Gubała, Jakub Mojsiejuk, Krzysztof Grochot, Jerzy Wrona, Jürgen Langer, Tianxiang Nan, Witold Skowroński

**Affiliations:** 1 Institute of Electronics, AGH University of Krakow, Al. Mickiewicza 30, 30-059 Kraków, Poland; 2 Singulus Technologies, 63796 Kahl am Main, Germany; 3 School of Integrated Circuit and Beijing National Research Center for Information Science and Technology (BNRist), Tsinghua University, Beijing, China

**Keywords:** Magnetic tunnel junctions, Perpendicular magnetic anisotropy, Ferromagnetic resonance, Random access memory, Electrical and electronic engineering, Applied physics, Electronic and spintronic devices

## Abstract

Magnetic tunnel junction (MTJ) used currently for data storage are characterized by perpendicular magnetic anisotropy, which is beneficial in terms of low current density required for switching and the thermal stability of the free magnetic layer. The other ferromagnet of MTJ, namely the reference layer is fixed using so-called synthetic antiferromagnetic (SAF) structure, which typically involves less-abundant material, such as Pt or Pd. We present an alternative stack structure, with the SAF based on Ni-Co superlattices, which is Pt-free. The reference layer of MTJ is characterized by the switching field above 250 mT. In MTJ nanopillars of diameter down to 80 nm, we show a robust switching with voltage pulses between 1 ms and 5 ns, tunneling magnetoresistance up to 140%, high thermal stability and switching current density of 2.6 MA/cm^2^. Our result show a promising route towards design of MTJs made of abundant materials.

## Introduction

Magnetic tunnel junction (MTJ) serves as the main building block of various types of electronic devices^1^, such as magnetic field sensor^2^ and magnetic random access memory cell^3^. The basic structure includes two ferromagnetic electrodes, separated by a tunneling barrier, which exhibit tunneling magnetoresistance (TMR) effect, i.e., resistance change depending on the relative orientation of two magnetization vectors. Due to the non-volatile character of magnetization, information can be stored in the magnetization direction, which does not require frequent refreshing^4^. Upon exciting with spin-polarized current (generated, for example, in another reference magnetic layer due to the spin filtering effect) due to the spin transfer torque (STT) effect^5,6^, the magnetization vector can be excited at the 100 ps timescale^7^, which can also be used to design high-frequency devices, such as generators and diodes^8,9^. Moreover, the non-linear character of the precession, tuned either by the current or magnetic field amplitudes^10^, can be further applied in neuromorphic computing platforms^11^. The large current can also switch the magnetization direction, which is the foundation for a memory writing process^12^. Contemporary MTJs are characterized by magnetic anisotropy perpendicular to the wafer plane^13,14^, which enhances thermal stability and reduces the critical current density needed for switching of the free magnetic layer. The other magnet, often referred to as the reference, typically requires a perpendicular synthetic antiferromagnetic (SAF) structure for magnetization vector stabilization, which involves Pt- or Pd-based materials to obtain a sufficiently large anisotropy field of a few hundreds of mT^15^. Recently, a Co-Pt-based SAF structure with an Ir spacer has been presented to show very high switching fields above 600 mT^16^. However, Pt and Pd are among the least abundant materials in the world, and therefore there is a need for an alternative way to create a reliable reference magnetic structure. The wafer-level study of the MTJ with FeCoB/Ru(Ta/Ru)/FeCoB-based SAF showed a promising route with a switching field of up to 130 mT^17^. Here, we present an alternative way to design the full MTJ structure which does not involve any less-abundant material. The reference layer is based on the Ni-Co superlattice^18^, coupled through a thin Ru spacer. The free and reference magnetic layers are based on the FeCoB alloy, with an additional atomically thin W interlayer^19^ within the synthetic free layer. The MTJ is characterized by the TMR up to 140%, thermal stability above 50 and low switching current density around 2.6 $\hbox {MA/cm}^2$.

## Results

**Fig. 1:**
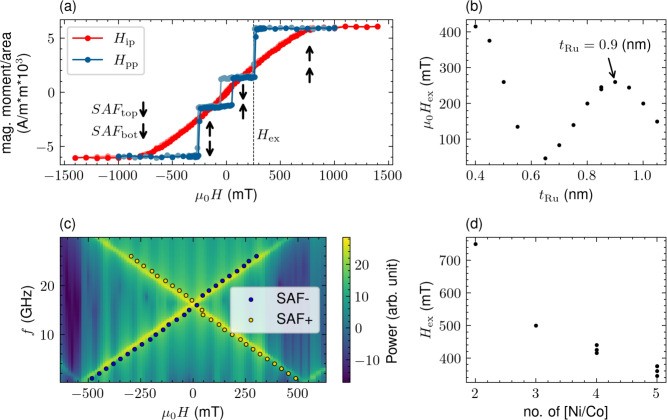
Optimization of synthetic antiferromagnetic (SAF). Magnetic moment per unit area vs. magnetic field applied in the sample plane ($H_\textrm{ip}$) and perpendicular to the sample plane ($H_\textrm{pp}$) for bottom part of the MTJ stack, i.e., buffer and SAF structure (**a**). Arrows indicate magnetic configuration in the given $H_\textrm{pp}$, $H_\textrm{ex}$ marks the exchange field value. Measured saturation magnetization of [Ni/Co] is 0.73 T. $H_\textrm{ex}$ as a function of Ru spacer thickness ($t_\textrm{Ru}$) with a second peak around $t_\textrm{Ru} = 0.9$ nm (**b**). Ferromagnetic resonance (FMR) of SAF structure measured at the given excitation frequency (*f*) with the magnetic field swept between +/- 400 mT (points). Heat map presents the simulation results for the system consisting of two antiferromagnetically coupled layers with the coupling energy $J_\textrm{ex} = - 180$ $\mu \hbox {J}/\hbox {m}^2$. $H_\textrm{ex}$ vs. number of [Ni/Co] layers in the top part of SAF (SAF$_\textrm{top}$) (**d**). Number of layers in the bottom part ($\hbox {SAF}_{\textrm{bot}}$) had little influence on the $H_\textrm{ex}$.

The MTJ structure studied here consists of the following layers (thicknesses in nm): buffer / SAF / FeCoB(1) / MgO(1) / FeCoB(1.3) / W(0.25)/ FeCoB(0.5)/ cap. The buffer is based on CuN/Ta layers to allow good electrical conductivity and smooth growth of MTJ^20^. SAF is made of two [Ni(0.6) / Co(0.25)] superlattices (six and three repetitions, respectively) with the Co(0.2) / Ru(0.9) / Co(0.2) spacer, which ensures strong antiferromagnetic coupling due to the RKKY-type interaction^21^. Figure 1 presents a summary of the optimization process of the SAF structure. Saturation magnetization of [Ni(0.6)/Co(0.25)] superlattice of $\mu _\textrm{0}M_\textrm{s} = 0.73$ T has been determined from the magnetometry measurements, in agreement with the previous reports^22,23^. The exchange field ($H_\textrm{ex}$) for the Ru thickness $t_\textrm{Ru} > 0.5$ nm (which ensures proper multilayer growth) is maximal for $t_\textrm{Ru} = 0.9$ nm. The magnetic anisotropy energy (*K*) of SAF layers have been determined from the ferromagnetic resonance (FMR) measurements by modeling *f*(*H*) dependence using cMTJ^24^. The broadband FMR was measured using different excitation frequencies between 3 and 24 GHz with the perpendicular magnetic field swept up to 0.9 T. The details of the measurement procedure are described in the Methods section. Finally, $H_\textrm{ex}$ has been optimized using different lamination layers of [Ni(0.6)/Co(0.25)], where strong (weak) influence of the number of layers in the top (bottom) SAF was found. Further details of the SAF optimization process are given in Methods section.

**Fig. 2:**
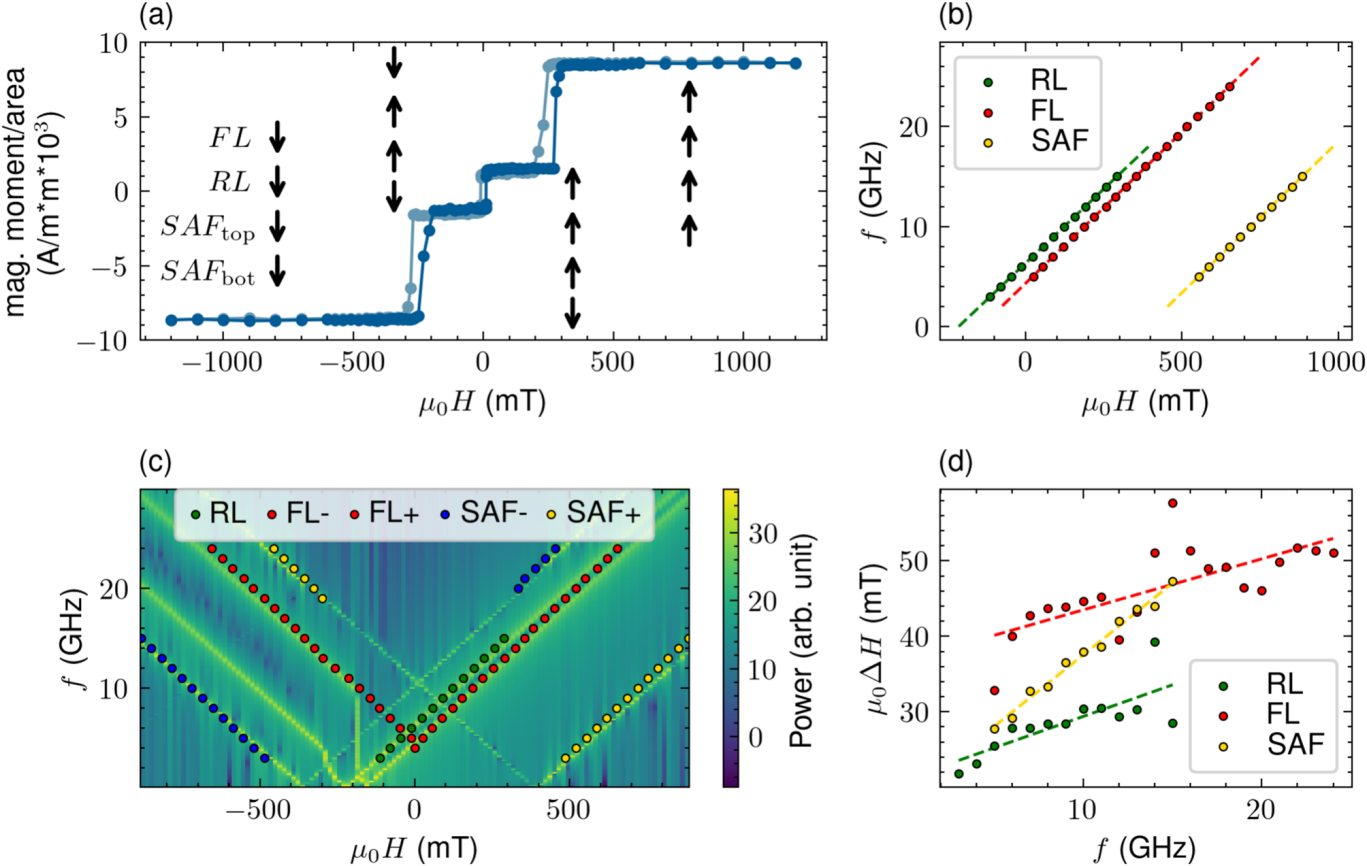
Wafer-level characterization of the optimized MTJ stack. Magnetic moment per unit area vs. $H_\textrm{pp}$ (**a**). Arrows indicate magnetic configuration of MTJ in the given $H_\textrm{pp}$, with the reference layer switching field above 250 mT. Measured saturation magnetization of FeCoB free layer (FL) is 1.12 T. After subtracting the magnetic moment of SAF, the saturation magnetization of FeCoB reference layer (RL) was calculated - 1.55 T. *f* vs. $H_\textrm{pp}$ for FL, RL and SAF enabled calculation of the magnetic anisotropy (**b**). Modeling of full FMR map is presented in (**c**). Every resonance mode is recreated in the simulation. Magnetization damping for each magnetic layer was calculated based on linewidth ($\Delta H$) vs. *f* dependence (**d**).

Full MTJ stack includes the texture break W(0.25) placed between SAF and FeCoB / MgO / FeCoB trialyer, which insures proper crystallization upon annealing at $370\,^\circ \hbox {C}$ of MTJ trilayer along [001] crystallographic direction^25^. MgO(0.9) / Ta(5) / Ru(5) capping is used to enhance the perpendicular magnetic anisotropy of the free layer^26,27^ and to protect mutlilayer from the degradation in the atmosphere. The thickness of the MgO tunnel barrier corresponds to the resistance area (RA) product of 10 $\hbox {Ohm}\times \mu \hbox {m}^2$, while the top MgO covered directly with Ta does not significantly influence resistance. Figure 2 presents the magnetometry and FMR results of the optimized full MTJ stack.

The saturation magnetization of the synthetic free layer (FeCoB(1.3)/ W(0.25)/FeCoB(0.5)) was calculated based on the measured magnetic moment and the assumption of the magnetic dead layer of 0.4 nm^28^. The saturation magnetization of the reference layer was calculated by subtracting the magnetic moment of SAF from the magnetic moment of MTJ (without the free layer). Values are summarized in Tab. 1. The multipeak resonance spectra can be analysed using the macrospin model, which allows for simulation of the FMR map depending on the magnetization saturation, magnetic anisotropy and damping of each layer. The magnetization damping was determined from the linewidth ($\Delta H$) dependence and is similar for FL and RL - around 0.01, typical for thin FeCoB^29^. Careful modeling of the FMR using the macrospin simulation package cMTJ allowed the calculation of anisotropy energies which are also gathered in Table 1. Both the reference and free layers are characterized by strong perpendicular magnetic anisotropy (PMA).

**Table 1:** Magnetic parameter of each layer determined from the vibrating sample magnetometry and ferromagnetic resonance.

Layer	$\mu _0\hbox {M}_S$	$\hbox {H}_{\textrm{k}}$	$\hbox {K}_{\textrm{u}}$	$\alpha$
	T	mT	$\hbox {kJ}/\hbox {m}^3$	unitless
SAF	0.73	400	260	0.026
RL	1.55	223	960	0.013
FL	1.12	145	563	0.011

**Fig. 3:**
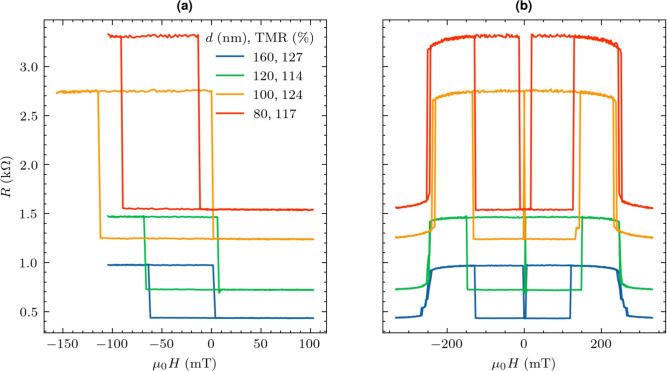
Resistance vs. perpendicular magnetic field dependence measured for MTJs of different diameters (**a**) for magnetic field values limited to the free layer coercivity. TMR ratio reaches 127% and the resistance are product is around 8 $\hbox {Ohm}\times \upmu \hbox {m}^2$. High-magnetic field sweeps shows the reference layer switching field of 250 mT (**b**).

After the analysis of the wafer-level parameters of the optimized MTJ we move on to the determination of the magnetotransport properties in the nm-scale devices. Using the microfabrication process MTJ nanopillars of diameters down to 80 nm were prepared. The selected devices were investigated in the magnetotransport measurement setup. The TMR versus the perpendicular field for devices of different diameters ranging from 300 down to 80 nm are shown in Fig. 3. The TMR exceeds 120% for the smallest device. The offset field increases with decreasing dimensions, due to the stray field interaction between the free and reference layers^30^. We note that the configuration of the SAF may also influence the asymmetry in the STT switch presented below^31^.

**Fig. 4:**
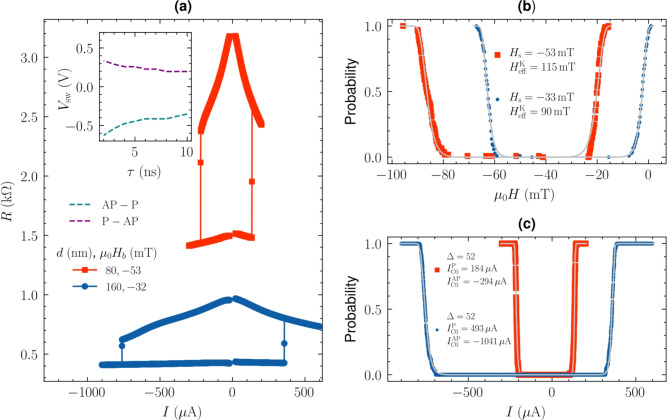
Current vs. resistance loops for MTJs of 160 and 80 nm diameter, measured using current pulses of 1 ms length and increasing amplitude, in the magnetic field corresponding to the offset field determined from TMR loop (**a**). Switching from antiparallel to parallel (from parallel to antiparallel) is measured for the current density of −3.1 $\hbox {MA/cm}^2$ (2.1 $\hbox {MA/cm}^2$). An inset presents the MTJ switching voltage amplitude as a function of the pulses length within the ns-range. The switching voltage increases with decreasing pulse duration and the probability is reduced for pulses shorter than 5 ns. The switching probability as a function of the magnetic field (**b**) and current pulses (**c**) repeated hundred times allowed for switching distribution determination and calculation of the thermal stability.

TMR measured in the high perpendicular magnetic field (Fig. 3b) indicates the switching of RL above 250 mT due to its coupling with Co-Ni-based SAF structure, similar value to the one measured on the wafer-level, which is sufficient for most applications^32^. Similarly, to the *R*(*H*) loop, the resistance vs. applied current pulses loops *R*(*I*) show a clear hysteresis - Fig. 4a. The average switching current density using the pulse length of 1 ms required to switch MTJ is 2.6 $\hbox {MA/cm}^2$. During the current-induced switching experiment, the static offset field was applied to compensate for the influence of the stray-field interactions. The thermal stability $\Delta$ presented in Fig. 4c of each device was calculated as follows. First, the offset $H_\textrm{s}$ and anisotropy $H_\textrm{k}^\textrm{eff}$ fields were determined by repeating the *R*(*H*) loop measurement 100 times and modeled using equation^33^:

1 $$\begin{aligned}&P = 1 - \exp \left( -\frac{\tau }{\tau _0} \exp \left( -\Delta \left( 1 - \frac{|H - H_\textrm{s}|}{H_\textrm{k}^\textrm{eff}} \right) ^{2} \right) \right) \end{aligned}$$

where $\tau$ is the field pulse width of 300 ms, $\tau _0$ is the inverse of the attempt frequency equal to 1 ns, $\Delta$ is the thermal stability. Then, with similar statistics we repeated *R*(*I*) loop with the magnetic field corresponding to the offset field. Finally, the switching probability as a function of the applied current (determined from the *R*(*I*) measurements) was modeled using Eq. 1 which allowed for the calculation of intrinsic switching current $I_\textrm{c0}$ and $\Delta$^34^:

2 $$\begin{aligned}&P = 2 - \exp \left( -\frac{\tau }{\tau _0} \exp \left( -\Delta \left( 1 - \frac{I}{I_{C0}^{P}} \right) \right) \right) - \exp \left( -\frac{\tau }{\tau _0} \exp \left( -\Delta \left( 1 - \frac{I}{I_{C0}^{AP}} \right) \right) \right) \end{aligned}$$

where $\tau$ is the current pulse width of 1 ms, *I* is the applied current, $I_\textrm{c0}^\mathrm {P(AP)}$ is the intrinsic switching current. The bias field equal to the offset field $H_\textrm{s}$, determined from the *R*(*H*) dependence was applied during the measurement - Fig. 4. The resulting stability $\Delta > 50$, which ensures the necessary data retention^4^. To observe the current-induced magnetization switching of the reference layer, a magnetic field of at least 200 mT is needed. We have verified that the thermal stability of the reference layer is even higher than for the free layer. Application of this compensating magnetic field between 30 and 50 mT (depending on the MTJ diameter) resulted in the switching current amplitude from the P to AP state being smaller than for the AP to P state, which is opposite to the STT efficiency model. However, we note that the magnetization reversal driven by STT is different than by the external magnetic field^35^, and the compensation of the magnetostatic interaction with the external magnetic field may additionally influence the magnetic domain structure and lead to the magnetization switching induced by the domain nucleation and propagation^33^. Also, second-order contributions to the magnetic anisotropy may additionally influence the stability diagram of the MTJ as a function of applied voltages and magnetic field^36^. In order to verify the current switching properties in the high-frequency regime, we repeated the switching experiment with short voltage pulses of $\tau \, <\, 10$ ns^37^. By applying a gradually increasing pulse amplitude, an increase in the average switching voltage from 300 mV to 600 mV was found (corresponding to the increase in the switching current density from 2.6 up to 5.2 MA/cm$^2$). For pulses shorter than 5 ns, the switching probability is reduced compared to longer pulses^38^, which is typical of the transition between thermally activated switching and precessional switching^39^.

## Discussion

The results indicate the applicability of using the Pt-free MTJ structure for memory devices with a sufficiently high switching field of the reference layer. Both the free-layer coercive field and the thermal stability are little affected by the MTJ diameter. For devices of 300 nm in diameter and above, the switching field is more scattered, which leads to multi-domain type switching. The smallest devices between 160 and 80 nm in diameter are characterized by a single domain switch, as a function of both the applied magnetic field and current pulses. Independent of the pillar size, the reference layer coercive field exceeds 250 mT, which shows promising routes toward Pt-free perpendicular MTJs. MTJs with slightly thicker free layer (1.4 nm FeCoB within the synthetic layer) are characterized by a higher TMR ratio (up to 145%), however, at the cost of reduced perpendicular magnetic anisotropy. Indeed, for thicker free layer squared hysteresis loop was measured only for the smallest diameters, due to stronger interaction of the stray field from the reference layer (not shown). In addition, the thermal stability for these devices is drops $\Delta < 25$. However, as expected, the switching field of the reference layer is not affected by the free layer parameters. For an optimal free-layer thickness of 1.3 nm FeCoB, the switching voltages of 600 mV at 5–10 ns long pulses, as well as the high thermal stability $\Delta > 50$ indicate readiness of the stack structure for memory applications.

## Summary

In conclusion, we propose a perpendicular magnetic tunnel junction multilayer stack, which does not contain any less abundant material, such as Pt or Pd. The synthetic antiferromagnet structure is based on two Co-Ni superlattices separated by a thin Ru spacer. The reference layer, which is strongly coupled to the SAF structure, is characterized by a high coercive field of 250 mT. The free layer, consisting of the synthetic FeCoB / W / FeCoB structure, is characterized by a strong perpendicular magnetic anisotropy $K_\textrm{u} = 563\hbox { kJ/m}^3$ and relatively low damping $\alpha$ = 0.011. After the microfabrication process, we verified a high tunneling magnetoresistance ratio above 130%, high thermal stability $\Delta > 50$ and low average switching voltage $V_\textrm{avg} = 0.35$ V (corresponding to the switching current density of $J_\textrm{C} = 2.6\,\hbox {MA/cm}^2$) for MTJ pillars down to 80 nm in diameter. The proposed stack structure may be used in future storage and sensor applications that require perpendicular magnetic anisotropy and abundant materials only during the deposition process.

## Methods

Magnetic tunnel junctions were deposited using a Singulus TIMARIS cluster tool sputtering system on thermally-oxidized Si wafers. After the deposition process, the samples were annealed at $370\,^\circ \hbox {C}$. The magnetic moment was measured using a vibrating sample magnetometer. The final SAF structure is a result of a detailed optimization process. The number of repetitions in the bottom and top SAF lamellas was varied between 2 and 7, and the thickness of Co was changed between 0 and 0.5 nm. The thickness of the Ru spacer was chosen for the second maximum of the antiferromagnetic RKKY-type coupling^40^. The final structure presented in the main text resulted in the highest switching field of the RL of 250 mT and magnetically compensated SAF. The TMR and the RA product were measured on the wafer-level using current in-plane tunneling (CIPT) and reached 140% and 10 $\hbox {Ohm}\times \mu \hbox {m}^2$. Magnetic anisotropy and damping were determined using a field-modulated ferromagnetic resonance measurement setup with a wafer placed face-down on the coplanar waveguide supplied with a sinusoidal signal of 12 dBm amplitude and frequency between 5 and 24 GHz. The output signal was measured with a zero bias broadband Schottky diode using a lock-in amplifier synchronized with the field modulation (384 Hz). Chips of different thicknesses of the free layer (between $t_\textrm{FL} = 1.3$ and 1.6 nm thick FeCoB within the synthetic free layer FeCoB($t_\textrm{FL}$)W(0.25)/FeCoB(0.5)) were fabricated using three-step, mix-match optical and electron beam lithography with circular cross-sectional pillars of diameter between 500 and 80 nm. The resistance-area product was calculated based on the dimensions determined using scanning electron microscope and the area-dependent resistance and yielded similar values to the one obtained using CIPT. A few tens of devices on each chip were measured with the TMR distribution between 110 and 135% for $t_\textrm{FL} = 1.3$ nm and up to 145% for $t_\textrm{FL} = 1.4$ nm. For $t_\textrm{FL} > 1.4$ nm we could not obtain sufficient PMA energy to measure the TMR ratio. Transport measurements were performed in the dedicated automatic probe station equipped with the perpendicular to plane magnetic field source and source-meters capable of measuring resistance vs. magnetic field and resistance vs. current pulses. ns-pulse switching characteristics were determined using pulse generator capable of voltage pulses application between 2 and 10 ns pulses length of programmable amplitude and the resistance measured using the source-meter after the pulse.

## Data Availability

All data included in this study are available upon request by contact with the corresponding author, Witold Skowroński (skowron@agh.edu.pl).

## References

[1] Kawahara, T., Ito, K., Takemura, R. & Ohno, H. Spin-transfer torque RAM technology: Review and prospect. *Microelectron. Reliab.* **52**, 613–627. 10.1016/j.microrel.2011.09.028 (2012).

[2] Cardoso, S. et al. Magnetic tunnel junction sensors with ptesla sensitivity. *Microsyst. Technol.* **20**, 793–802. 10.1007/s00542-013-2035-1 (2014).

[3] Ikeda, S. et al. Magnetic tunnel junctions for spintronic memories and beyond. *IEEE Trans. Electron Devices* **54**, 991–1002. 10.1109/TED.2007.894617 (2007).

[4] Ikegawa, S., Mancoff, F. B., Janesky, J. & Aggarwal, S. Magnetoresistive random access memory: Present and future. *IEEE Trans. Electron Devices* **67**, 1407–1419. 10.1109/TED.2020.2965403 (2020).

[5] Slonczewski, J. C. Current-driven excitation of magnetic multilayers. *J. Magn. Magn. Mater.* **159**, L1 (1996).

[6] Berger, L. Emission of spin waves by a magnetic multilayer traversed by a current. *Phys. Rev. B* **54**, 9353 (1996). 10.1103/physrevb.54.9353 9984672

[7] Zhang, C., Takeuchi, Y., Fukami, S. & Ohno, H. Field-free and sub-ns magnetization switching of magnetic tunnel junctions by combining spin-transfer torque and spin-orbit torque. *Appl. Phys. Lett.* **118**, 092406. 10.1063/5.0039061 (2021).

[8] Harder, M., Gui, Y. & Hu, C.-M. Electrical detection of magnetization dynamics via spin rectification effects. *Phys. Rep.* **661**, 1–59. 10.1016/j.physrep.2016.10.002 (2016).

[9] Kiselev, S. I. et al. Microwave oscillations of a nanomagnet driven by a spin-polarized current. *Nature* **425**, 380 (2003). 14508483 10.1038/nature01967

[10] Kim, J.-V. Chapter four - spin-torque oscillators. In Camley, R. E. & Stamps, R. L. (eds.) *Solid State Physics*, vol. 63 of *Solid State Physics*, 217–294, 10.1016/B978-0-12-397028-2.00004-7(Academic Press, 2012).

[11] Romera, M. et al. Vowel recognition with four coupled spin-torque nano-oscillators. *Nature* **563**, 230–234. 10.1038/s41586-018-0632-y (2018). 30374193 10.1038/s41586-018-0632-y

[12] Lee, T. Y. *et al.* Advanced mtj stack engineering of stt-mram to realize high speed applications. In *2020 IEEE International Electron Devices Meeting (IEDM)*, 11.6.1–11.6.4, 10.1109/IEDM13553.2020.9372015(2020).

[13] Ikeda, S. et al. A perpendicular-anisotropy CoFeB-MgO magnetic tunnel junction. *Nat. Mater.* **9**, 721. 10.1038/nmat2804 (2010). 20622862 10.1038/nmat2804

[14] Worledge, D. C. et al. Spin torque switching of perpendicular Ta/CoFeB/MgO-based magnetic tunnel junctions. *Appl. Phys. Lett.* **98**, 022501. 10.1063/1.3536482 (2011).

[15] Mangin, S. et al. Current-induced magnetization reversal in nanopillars with perpendicular anisotropy. *Nat. Mater.* **5**, 210–215. 10.1038/nmat1595 (2006).

[16] Yakushiji, K., Sugihara, A., Fukushima, A., Kubota, H. & Yuasa, S. Very strong antiferromagnetic interlayer exchange coupling with iridium spacer layer for perpendicular magnetic tunnel junctions. *Appl. Phys. Lett.* **110**, 092406. 10.1063/1.4977565 (2017).

[17] Cuchet, L. et al. Perpendicular magnetic tunnel junctions with a synthetic storage or reference layer: A new route towards pt- and pd-free junctions. *Sci. Rep.* **6**, 21246. 10.1038/srep21246 (2016). 26883933 10.1038/srep21246 PMC4756698

[18] Jungblut, R., Johnson, M. T., Aan de Stegge, J., Reinders, A. & Broeder, F. J. A. Orientational and structural dependence of magnetic anisotropy of Cu/Ni/Cu sandwiches: Misfit interface anisotropy. *J. Appl. Phys.* **75**, 6424–6426. 10.1063/1.355372 (1994).

[19] Skowroński, W. et al. Understanding stability diagram of perpendicular magnetic tunnel junctions. *Sci. Rep.* **7**, 10172 (2017). 28860571 10.1038/s41598-017-10706-2 PMC5579061

[20] Banasik, M. et al. Magnetic properties and magnetization dynamics of magnetic tunnel junction bottom electrode with different buffer layers. *IEEE Trans. Magn.* **51**, 1–4. 10.1109/TMAG.2015.2440561 (2015).26203196

[21] Parkin, S. S. P., More, N. & Roche, K. P. Oscillations in exchange coupling and magnetoresistance in metallic superlattice structures: Co/ru, co/cr, and fe/cr. *Phys. Rev. Lett.* **64**, 2304 (1990). 10041640 10.1103/PhysRevLett.64.2304

[22] Arora, M. et al. Magnetic properties of co/ni multilayer structures for use in STT-RAM. *J. Phys. D Appl. Phys.* **50**, 505003. 10.1088/1361-6463/aa97fa (2017).

[23] Girod, S. et al. Strong perpendicular magnetic anisotropy in ni/co(111) single crystal superlattices. *Appl. Phys. Lett.* **94**, 262504. 10.1063/1.3160541 (2009).

[24] Mojsiejuk, J., Ziętek, S., Grochot, K., Skowroński, W. & Stobiecki, T. Simulation package for analysis of multilayer spintronic devices. *NPJ Comput. Mater.* **9**, 54. 10.1038/s41524-023-01002-x (2023).

[25] Yuasa, S. & Djayaprawira, D. D. Giant tunnel magnetoresistance in magnetic tunnel junctions with a crystalline MgO (0 0 1) barrier. *J. Phys. D Appl. Phys.* **40**, R337 (2007).

[26] Thomas, L. et al. Perpendicular spin transfer torque magnetic random access memories with high spin torque efficiency and thermal stability for embedded applications (invited). *J. Appl. Phys.* **115**, 172615. 10.1063/1.4870917 (2014).

[27] Kubota, H. et al. Enhancement of perpendicular magnetic anisotropy in FeB free layers using a thin MgO cap layer. *J. Appl. Phys.* **111**, 07C723. 10.1063/1.3679393 (2012).

[28] Skowroński, W. et al. Underlayer material influence on electric-field controlled perpendicular magnetic anisotropy in CoFeB/MgO magnetic tunnel junctions. *Phys. Rev. B* **91**, 184410. 10.1103/PhysRevB.91.184410 (2015).

[29] Liu, X., Zhang, W., Carter, M. J. & Xiao, G. Ferromagnetic resonance and damping properties of cofeb thin films as free layers in mgo-based magnetic tunnel junctions. *J. Appl. Phys.* **110**, 033910. 10.1063/1.3615961 (2011).

[30] Jiancheng, H. et al. Effect of the stray field profile on the switching characteristics of the free layer in a perpendicular magnetic tunnel junction. *J. Appl. Phys.* **117**, 17B721. 10.1063/1.4916037 (2015).

[31] Lavanant, M. et al. Asymmetric magnetization switching in perpendicular magnetic tunnel junctions: Role of the synthetic antiferromagnet’s fringe field. *Phys. Rev. Appl.* **11**, 034058. 10.1103/PhysRevApplied.11.034058 (2019).

[32] Dieny, B. et al. Impact of external magnetic fields on stt-mram: An application note. *IEEE Electron Devices Magazine* **2**, 52–59. 10.1109/MED.2024.3442086 (2024).

[33] Li, Z. & Zhang, S. Thermally assisted magnetization reversal in the presence of a spin-transfer torque. *Phys. Rev. B*10.1103/PhysRevB.69.134416 (2004).

[34] Sato, H. et al. Perpendicular-anisotropy CoFeB-MgO magnetic tunnel junctions with a MgO/CoFeB/Ta/CoFeB/MgO recording structure. *Appl. Phys. Lett.* **101**, 022414. 10.1063/1.4736727 (2012).

[35] Myers, E. B. et al. Thermally activated magnetic reversal induced by a spin-polarized current. *Phys. Rev. Lett.* **89**, 196801. 10.1103/PhysRevLett.89.196801 (2002). 12443138 10.1103/PhysRevLett.89.196801

[36] Strelkov, N. et al. Stability phase diagram of a perpendicular magnetic tunnel junction in noncollinear geometry. *Phys. Rev. B* **95**, 184409. 10.1103/PhysRevB.95.184409 (2017).

[37] Huai, Y. et al. High performance perpendicular magnetic tunnel junction with co/ir interfacial anisotropy for embedded and standalone stt-mram applications. *Appl. Phys. Lett.* **112**, 092402. 10.1063/1.5018874 (2018).

[38] Bedau, D. et al. Spin-transfer pulse switching: From the dynamic to the thermally activated regime. *Appl. Phys. Lett.* **97**, 262502. 10.1063/1.3532960 (2010).

[39] Apalkov, D. et al. Spin-transfer torque magnetic random access memory (STT-MRAM). *J. Emerg. Technol. Comput. Syst.*10.1145/2463585.2463589 (2013).

[40] Bandiera, S. et al. Comparison of synthetic antiferromagnets and hard ferromagnets as reference layer in magnetic tunnel junctions with perpendicular magnetic anisotropy. *IEEE Magn. Lett.* **1**, 3000204–3000204. 10.1109/LMAG.2010.2052238 (2010).

